# Development and Evaluation of Cellulosic Esters Solvent Removal-Induced In Situ Matrices for Loading Antibiotic Drug for Periodontitis Treatment

**DOI:** 10.3390/polym17111551

**Published:** 2025-06-02

**Authors:** Ei Mon Khaing, Napaphol Puyathorn, Nuttapon Yodsin, Nakharin Phonarwut, Warakon Thammasut, Catleya Rojviriya, Wiwat Pichayakorn, Supanut Phattarateera, Thawatchai Phaechamud

**Affiliations:** 1 Program of Pharmaceutical Engineering, Department of Industrial Pharmacy, Faculty of Pharmacy, Silpakorn University, Nakhon Pathom 73000, Thailand; khaing_e@silpakorn.edu (E.M.K.); thammasut_w@silpakorn.edu (W.T.); 2 Department of Pharmaceutical Sciences, Faculty of Pharmacy, Chiang Mai University, Chiang Mai 50200, Thailand; napaphol.p@cmu.ac.th; 3 Center of Excellence in Pharmaceutical Nanotechnology, Chiang Mai University, Chiang Mai 50200, Thailand; 4 Department of Chemistry, Faculty of Science, Silpakorn University, Nakhon Pathom 73000, Thailand; yodsin_n@su.ac.th; 5 Department of Chemistry and Center of Excellence for Innovation in Chemistry, Faculty of Science, Ubon Ratchathani University, Ubon Ratchathani 34190, Thailand; nakhalin.ph.65@ubu.ac.th; 6 Synchrotron Light Research Institute, Nakhon Ratchasima 30000, Thailand; catleya@slri.or.th; 7 Department of Pharmaceutical Technology, Faculty of Pharmaceutical Sciences, Prince of Songkla University, Songkhla 90110, Thailand; wiwat.p@psu.ac.th; 8 Plastic Technology Research Team, Advanced Polymer Research Group, National Metal and Materials Technology Center (MTEC), Pathum Thani 12120, Thailand; supanut.pha@mtec.or.th; 9 Department of Industrial Pharmacy, Faculty of Pharmacy, Silpakorn University, Nakhon Pathom 73000, Thailand

**Keywords:** in situ matrices, cellulosic esters, antibiotic drug, periodontitis

## Abstract

Cellulose acetate butyrate (CAB) and cellulose acetate propionate (CAP) are biobased materials that are insoluble in water and present a potential alternative to fossil-based plastics. Solvent removal-induced in situ matrices are gaining attention as an innovative dosage form for localized drug delivery for periodontitis therapy. This study aims to develop levofloxacin hemihydrate (Lh)-loaded in situ matrices formed through solvent removal, incorporating various molecular weights (MWs) and concentrations of CAB and CAP. Increased MWs and higher concentrations of these cellulosic esters significantly improved formulation viscosity and injection force, contributing to enhanced phase inversion and greater matrix toughness. Microscopic analysis of interfacial phase changes revealed progressive thickening of the matrix over time, which was influenced by polymer concentration and limited solvent movement. The transformed matrices with high MW CAP and elevated CAB content demonstrated prolonged drug release, predominantly following first-order kinetics, suggesting drug dissolution and diffusion through the scaffold structure. CAB-based in situ matrices containing 15% and 20% polymer exhibited low viscosities suitable for injection, along with optimal gel formation for maintaining their shape, and adhered effectively to periodontal pockets. These matrices provided extended Lh release for up to 120 h and inhibited the growth of periodontopathic bacteria for over 15 days. Therefore, the developed Lh-loaded in situ matrices show promise as an effective treatment for periodontitis, warranting further research to explore their therapeutic potential.

## 1. Introduction

**Figure 1:**
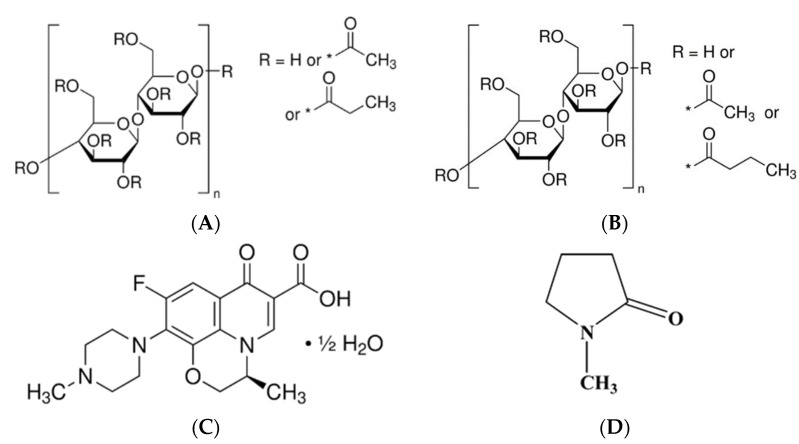
Chemical structures of cellulose acetate propionate (**A**), cellulose acetate butyrate (**B**), levofloxacin hemihydrate (**C**), and *N*-methyl pyrrolidone (NMP) (**D**). * substitution group.

Cellulose acetate butyrate (CAB) and cellulose acetate propionate (CAP) are significant polymers in bioplastic applications [1]. Furthermore, these polymers readily dissolve in most common solvents, such as acetone, produce transparent cast films, and exhibit excellent water resistance [1,2,3]. As raw materials derived from renewable resources, they are potential candidates for replacing fossil-based materials [3]. Compared to monocellulose esters, like cellulose acetate, CAB and CAP offer several advantages, including superior solubility, structural stability, resistance to light and weather, excellent leveling properties, high gloss retention, and remarkable transparency [4]. Controlled theophylline release was achieved by encapsulating it within CAP microspheres using the emulsion–solvent evaporation method [5]. Ketotifen release from CAB membranes is higher, whereas CAP membranes provide a more uniform release. Both membranes effectively enable the controlled release of ketotifen, attributed to their regulated swelling behavior [6]. The chemical structures of CAP and CAB are illustrated in Figure 1A and Figure 1B, respectively.

Solvent removal-induced in situ matrices are currently utilized as a promising delivery system for localized drug targeting, such as in periodontal pockets for the treatment of periodontitis [7]. Injecting an aqueous-insoluble matrix former solution into an aqueous environment, such as an aqueous fluid in a periodontal pocket, can provoke solvent removal from the formulation. The water-insoluble characteristics of CAP and CAB make them suitable candidates for use as matrix-forming agents in in situ delivery systems. The solvent removal process can promote the formation of CAP and CAB matrices, which in turn helps regulate the release of the incorporated drug [8]. The primary pathogens responsible for periodontitis are anaerobic *Gram*-negative bacteria. However, during the aggressive stage, the invasion of other bacterial species and yeast may also be observed [9]. Currently, periodontal disease affects approximately 20–50% of the global population, with around 10.8% showing signs of advanced chronic periodontitis [10]. According to the Global Burden of Disease Study, periodontal disease ranks as the 11th most prevalent condition worldwide. The primary contributors to periodontitis have been identified as bacterial biofilm formation on tooth surfaces and inflammation within the periodontal pocket [8]. Age and smoking have been identified as potential factors associated with the prevalence of periodontitis among patients with diabetes mellitus [11]. Eradicating these accumulated microbes through systemic antibacterial drug administration carries a risk of side effects. In the treatment of periodontitis, the periodontal pocket is typically cleaned by dental professionals using an irrigating solution. Antimicrobial agents, such as chlorhexidine mouthwash, are commonly used to flush the area and reduce the bacterial plaque load. Due to the limited accessibility of deep periodontal pockets and adjacent infected tissues, which are often difficult to reach, antibacterial treatments are commonly employed as adjuncts to conventional mechanical procedures, such as scaling and root planning [7]. However, systemic antibiotic administration whether through oral dosage forms, hydrogels, or mouthwashes has notable limitations, particularly in achieving and maintaining effective drug concentrations at the site of infection due to rapid drug clearance and short duration of action [12]. Local antimicrobial delivery systems, such as solvent removal-induced in situ forming matrices, offer an alternative approach. These systems enable the direct application of therapeutic agents into the periodontal pocket, aiming to sustain effective drug levels over time while reducing systemic side effects for improved patient compliance due to reduced drug administration frequency [8]. Therefore, delivering antibacterial drugs locally to the periodontal pocket for treatment presents an appealing alternative.

Levofloxacin has been shown to markedly enhance clinical and microbiological indicators in patients with chronic periodontitis after oral administration by reducing the proportion of sites that tested positive for periodontopathic bacteria [12]. Levofloxacin HCl-loaded in situ matrices have been reported, utilizing zein-based formulations in dimethyl sulfoxide and glycerol formal [13] as well as Eudragit L-based formulations in monopropylene glycol [14] as solvents. Levofloxacin hemihydrate (Lh) (Figure 1C) is a broad-spectrum antibiotic classified as a third-generation fluoroquinolone. It is the levo isomer of ofloxacin and exhibits water solubility of up to 25 mg/mL [15]. The investigation of Lh-loaded solvent removal-induced CAB- and CAP-based in situ matrices has not been reported in prior studies.

This study aimed to develop and evaluate Lh-loaded in situ matrices using N-methyl pyrrolidone (NMP) (Figure 1D) as a solvent. The formulation employed a solvent removal mechanism, with CAB and CAP as the matrix-forming agents, for the targeted treatment of periodontitis. To accomplish this, the matrix-forming behavior of CAB and CAP was examined at various concentrations. The effects of these cellulosic ester polymer types and concentrations were then evaluated and discussed in relation to their physicochemical properties, matrix formation, drug release, matrix morphology, and antimicrobial activities of Lh-loaded solvent removal-induced CAB- and CAP-based in situ matrices. These evaluations are designed to validate the formulation’s effectiveness, thereby improving therapeutic outcomes in drug delivery systems.

## 2. Materials and Methods

### 2.1. Materials

Four distinct types of cellulose-based polymers were employed as matrix-forming agents for the in situ matrix formulations: two grades of cellulose acetate butyrate (CAB) with different substitution patterns and two types of cellulose acetate propionate (CAP). The CAB variants examined included CAB 551-0.01 (A), which had a butyryl content of 52% *w*/*w*, an acetyl content of 2% *w*/*w*, and a molecular weight (MW) of 16,000 Daltons, and CAB 381-0.1 (B), with a butyryl content of 38% *w*/*w*, an acetyl content of 13.5% *w*/*w*, and an MW of 20,000 Daltons. The CAP grades used were CAP 482-0.5 (C), containing a propionate content of 45% *w*/*w*, an acetyl content of 1.5% *w*/*w*, and an MW of 25,000 Daltons, as well as CAP 504-0.2 (D), characterized by acetyl and propionyl contents of 1.0% *w*/*w* and 42.5% *w*/*w*, respectively, and an MW of 15,000 Daltons. All polymers were attained from Eastman Chemical Sdn. Bhd. (Kuantan, Malaysia).

Levofloxacin hemihydrate (Lh) (Lot No. ZF07U2308002), obtained from T.O. Chemicals (1979) Co., Ltd., Pathumthani, Thailand, was utilized as the antimicrobial agent. NMP (Lot No. 144560-118, QReC, Auckland, New Zealand) was utilized as the solvent. Phosphate-buffered saline (PBS) (pH 6.8) was prepared by dissolving potassium dihydrogen orthophosphate and sodium hydroxide (Ajax FineChem, Wollongong, New South Wales, Australia) in water. Agarose (Lot No. H7014714) from Vivantis, Selangor Darul Ehsan, Malaysia, was used to prepare the agarose gel. Sodium fluorescein (Lot No. SHBL6563) and Nile red (Lot No. BCBP8959V), both obtained from Sigma-Aldrich, Inc., Louis, MO, USA, were employed for investigating interfacial interactions.

The microbial strains evaluated as periodontal pathogens included *Staphylococcus aureus* ATCC 6538 and *Candida albicans* ATCC 10231, both provided by the Ministry of Public Health, Thailand, and *Porphyromonas gingivalis* ATCC 33277, sourced from Thai Can Biotech, Thailand. For antibacterial testing, *S. aureus* was cultured using Tryptic Soy Agar (TSA) and Tryptic Soy Broth (TSB), while *C. albicans* was grown on Sabouraud Dextrose Agar (SDA) and in Sabouraud Dextrose Broth (SDB). These culture media were obtained from Difco, Detroit, MI, USA. Anaerobic antibacterial testing against *P. gingivalis* was conducted using sheep blood agar, supplied by M&P Impex, Bangkok, Thailand.

### 2.2. Preparation of In Situ Matrix Systems

Two different grades of CAB and CAP were separately weighed at concentrations of 5%, 10%, 15%, and 20% (*w*/*w*), and each was dissolved in NMP. The solutions were prepared by a mixing method with continuous agitation using a magnetic stirrer for 24 h. The composition of the formulations is summarized in Table 1. Following this, 1% *w*/*w* Lh was dissolved in the selected polymeric solutions as specified in Table 1. The control groups consisted of polymer-free solution (LhN) and NMP

### 2.3. Evaluations

#### 2.3.1. Physical Appearance and Viscosity Measurement

All formulations were assessed for their physical characteristics focusing on clarity, color, and the occurrence of precipitates. The viscosity test was conducted using a cone-plate viscometer (RM 100 CP2000 Plus, Lamy Rheology Instruments, Champagne-au-Mont-d’Or, France) at a controlled temperature of 25 °C. Measurements were recorded at 15 s intervals using a 10 mm diameter cone in conjunction with a 70 mm bottom plate. To ensure uniform comparison across the formulations, a consistent shear rate of 120 rpm was applied during the measurements. These viscosity evaluations were undertaken in triplicate.

#### 2.3.2. Injectability Test

Each 1 mL formulation was drawn into a 1 mL plastic syringe fitted with a 21-gauge needle and placed on a stainless-steel stand of texture analyzer (TA.XT Plus, Stable Micro Systems, Godalming, UK). The upper probe of the instrument moved downward at a speed of 1.0 mm/s, applying a force of 0.1 N to the base of the syringe barrel [8,16]. The highest compression force was measured to indicate injectability. A low injection force value indicates that the formulation can be administered more easily through the needle. Each test was performed in triplicate (*n* = 3).

#### 2.3.3. Determination of Contact Angle

Contact angle measurements were undertaken employing a drop shape analyzer (FTA 1000, First Ten Angstroms, Newark, CA, USA) through the sessile drop approach. Experiments were conducted on both agarose gel and glass slide surfaces, using a droplet releasing rate of 1.9 µL/s through a 14-gauge needle, in accordance with previously published protocols [16]. Measurements were recorded at 5 s after droplet application, and each test was performed in triplicate.

#### 2.3.4. Study of Mechanical Property

The mechanical properties of the in situ matrices were assessed following a solvent removal process in a 7 mm diameter hollow agarose gel containing PBS (pH 6.8) over a 3-day period [16,17]. A texture analyzer as mentioned above was used for this test. A stainless-steel cylindrical probe with a diameter of 5 mm was inserted into the center of the cellulose ester matrix [17]. The force–displacement profile was recorded over time, enabling the determination of both the maximum penetration force and the residual adhesion force (remaining force) (*n* = 3).

#### 2.3.5. Test of Gel Formation in an Aqueous Environment

To check the phase transformation process induced by solvent removal, which triggers polymeric separation, the change from a solution to a gel or solid matrix-like mass was assessed. The 1 mL of the prepared formula was injected directly into 5 mL of PBS, pH 6.8, using an 18-gauge stainless-steel needle. Morphological changes were documented at 5 min intervals to observe the progression of the phase transformation. The change characteristic from solution to gel from solvent removal was also assessed by injecting 150 µL of the in situ matrix formulations into a hollow agarose well. Changes in the cross-section of the in situ matrix systems within the agarose well were captured using a stereo microscope (SMZ 171 Series, Motic Inc., Kowloon, Hong Kong) at 5 min. To mimic the environment of the periodontal crevicular pocket, this hollow agarose well model was developed. A 0.6% agarose solution was prepared by dissolving agarose in a simulated saliva medium (phosphate-buffered saline, PBS, pH 6.8). This solution was then cast into Petri dishes to form a gel with a thickness of approximately 1 cm. After solidification, a central cylindrical cavity measuring 7 mm in diameter and with a volume of 300 μL was created using a cylindrical mold for filling the test formulation as described above.

#### 2.3.6. Microscopic Interfacial Change

Interfacial interaction between the formulation and the surrounding aqueous medium within the agarose was examined to understand phase transformation by employing a technique modified from earlier research [17]. The agarose gel was placed adjacent to a 20 µL dosage form near its incision edge. Phase transitions induced by exposure to an agarose rim were checked under an inverted stereomicroscope (Nikon Eclipse TE2000S, Nikon, Kawasaki, Japan) at 5 min.

To check the dissipation of aqueous and NMP during phase inversion, fluorescent tracking was conducted using a hydrophilic compound (sodium fluorescein) and a hydrophobic compound (Nile red), following procedures established in earlier research [18]. The used conditions involved a 0.2 μg/mL sodium fluorescein-loaded agarose gel in contact with a 0.4 μg/mL Nile red-loaded formulation. Fluorescent color variation of both sides was recorded using an inverted fluorescent microscope (Nikon Eclipse TE2000S, Nikon, Kawasaki, Japan) with a 40× magnification. The green fluorescence of sodium fluorescein was visualized using a blue (B2A) filter with excitation at 450–490 nm, while the red fluorescence of Nile red was monitored with a green (G2A) filter, excited at 510–560 nm. Images were captured at 1, 3, 5, 7, and 10 min.

#### 2.3.7. Drug Content and In Vitro Drug Release Test

In vitro drug release experiments were carried out over a duration of 120 h to analyze the release pattern of Lh from the formulations using the cup method. A cylindrical porcelain cup, with a diameter of 1 cm and height of 1.2 cm, was loaded with 0.3 g of the formulation and submerged in 80 mL of PBS (pH 6.8) at 37 °C and a rotational speed of 50 rpm. At designated time intervals, 5 mL samples of the release medium were collected, and 5 mL of fresh new PBS was added. The drug content in both the formulation and the released drug concentration was measured using a UV–visible spectrophotometer (Cary 60 UV-Vis, Model G6860A, Agilent, Selangor, Malaysia) at a wavelength of 287 nm. NMP-absorbed UV light below 200 nm did not affect Lh detection. Prior to analysis, the samples were passed through a 0.45 µm nylon membrane filter. All experiments were performed in six independent replicates for drug content in formulation determination and in triplicate for drug release in released fluid. The cumulative drug released was calculated and plotted against the time point of sampling.

The drug release mechanism was assessed by fitting the dissolution data to several mathematical models, including zero-order, first-order, Higuchi’s, Korsmeyer–Peppas, and Peppas–Sahlin models. A better model fit was indicated by higher coefficients of determination (R^2^), lower Akaike Information Criterion (AIC) values, and increased Model Selection Criterion (MSC) scores. The analysis was conducted using DD-Solver^®^ version 1, a Microsoft Excel add-in (Redmond, WA, USA) developed with Visual Basic for Applications [18].

#### 2.3.8. In Vitro Degradability Study

The prepared formulations were precisely weighed, transferred into porcelain cups, and then subjected to the same conditions as the drug release study. After 120 h, the remaining matrix was collected, dried in a conventional hot air oven for 48 h, and stored in a desiccator prior to final weighing. The % weight loss was calculated relative to initial weight (*n* = 3).

#### 2.3.9. Topography of Transformed In Situ Matrices

##### Topography Observation by a Scanning Electron Microscope (SEM)

An SEM photograph was employed to compare the morphology of the prepared formulations after solvent removal, offering high-resolution visualization of their microstructural surface properties. After a 120 h drug release period, the remnants were thoroughly washed several times with distilled water to remove any residual solvent. The washed remnants were subsequently dried using a desiccant for two weeks to ensure thorough dehydration prior to SEM analysis.

Once completely dried, the samples were coated with a thin gold layer using a sputtering device (BIO-RAD SEM Coating Unit PS3, BIO-RAD Laboratories Ltd., Hercules, CA, USA). The gold-coated samples were then analyzed using a field emission scanning electron microscope (TESCAN MIRA3, Brno-Kohoutovice, Czech Republic) at magnifications of 30×, 200×, 1000×, and 10,000×, with an accelerating voltage of 15 kV. Additionally, the morphology of intact CAB, CAP, and Lh powders was examined using scanning electron microscopy (SEM) at magnifications of 200× and 1000× to observe their individual characteristics.

##### Topography Observation by Synchrotron Radiation X-Ray Tomographic Microscopy (SRXTM)

SRXTM was utilized to analyze the internal and surface morphology of the specimens in three dimensions. This non-destructive, high-resolution imaging method enabled detailed observation of microstructural characteristics, including phase distribution, pore structure, and density variations within the material. The remnants from the above drying process in Section 2.3.9 were further scrutinized using SRXTM (detection setup comprising a 200 µm thick YAG scintillator (Crytur, Turnov, Czech Republic) at the Synchrotron Light Research Institute (SLRI) beamline in Nakhon Ratchasima, Thailand, to obtain comprehensive insights into their three-dimensional internal structure). The prepared specimens were subsequently analyzed for their three-dimensional porosity through segmentation analysis with Octopus Analysis (TESCAN, Gent, Belgium) [19].

#### 2.3.10. Antimicrobial Activity Test

The bioactivity was assessed by evaluating the antimicrobial effectiveness of formulations against *S. aureus* ATCC 6538, *C. albicans* ATCC 10231, and *P. gingivalis* ATCC 33277 using the agar diffusion assay (cylinder plate method), as per a previously described procedure [8]. A 100 µL aliquot of each formulation was dispensed into cylinder caps placed on the respective agar plates. Plates for *S. aureus* and *C. albicans* were incubated at 37 °C in a standard incubator (Thermo Scientific Precision Compact Incubators, Thermo Scientific, Cincinnati, OH, USA), whereas *P. gingivalis* plates were incubated in an anaerobic container at the same temperature. LhN and NMP were employed as the control groups. After 24 h of incubation, the inhibition zones were measured for their diameters (*n* = 3).

Furthermore, the extended antimicrobial activity of the in situ matrix formulations was evaluated by transferring the formulation-loaded cup into freshly inoculated agar media at regular intervals. This process was repeated on days 1, 7, and 15 to monitor any changes in the diameter of the inhibition zones against *S. aureus, C. albicans*, and *P. gingivalis* (*n* = 3).

### 2.4. Statistical Analysis

The obtained data were expressed as the mean ± standard deviation (S.D.), with statistical significance set at *p* < 0.05. Data analysis began with a one-way analysis of variance (ANOVA) to identify overall differences among the groups. A post hoc least significant difference (LSD) test was then conducted to determine specific group comparisons. The statistical evaluation was run using SPSS software for Windows (version 11.5).

## 3. Results and Discussion

### 3.1. Physical Appearance, Viscosity, and Injectability

All prepared formulations appeared as clear, transparent solutions without any precipitation. The viscosity and injectability force values increased with higher concentrations of CAB and CAP, as detailed in Table 2. Among the formulations, those containing CAB type A exhibited lower viscosity and injectability force values compared to CAB type B, CAP type D, and CAP type C, respectively. Consequently, the type and concentration of these cellulosic esters significantly influenced the physical properties of the drug-loaded solutions [20].

The lower viscosity of CAB type A can be attributed to its relatively lower molecular weight of 16 kDa compared to CAB type B, as described earlier in Section 2.1. Interestingly, CAP type C demonstrated a higher molecular weight than CAP type D [20]. The slightly increased viscosity of LhN compared to NMP is likely due to the reduced solvent content in the system.

The ratio of acetyl to butyryl substitution in CAB and acetyl to propionyl substitution in CAP can be adjusted during synthesis, leading to variations in their properties [3]. CAB dissolves easily in most bulk solvents, forms transparent cast films, and exhibits resistance to water [2,21]. Generally, a good solvent for polymer dissolution can reduce the formulation’s viscosity due to the dominance of polymer–solvent interactions over polymer–polymer interactions [22]. Additionally, polymers tend to adopt different chain configurations in various dispersing fluids, depending on their affinities with the solvents [22,23].

In dosage form design, in situ matrices are administered via targeted injection into the periodontal pocket; thus, the formulations should exhibit a low-viscosity fluid state to allow for painless injection [7,8]. Typically, a higher expulsion force indicates reduced injectability. The work required to expel the formulation through needles, representing its injectability (as shown in Table 2), increased in alignment with the viscosity results. In practice, the total expulsion force for each solution was less than 50 N·mm, meeting the acceptability criteria for injection [13,14].

### 3.2. Contact Angel

The spreadability of the prepared formulations, measured through their contact angle values on glass slides and agarose surfaces, is summarized in Table 2. The transformation of a solution into a gel state and subsequently into a solid, matrix-like mass was examined on agarose surfaces to simulate the conditions of the gingival mucosa within periodontal pockets. On the dry surface of the glass slide, the contact angle increased progressively with higher concentrations of cellulosic esters, directly correlating with the viscosity of the formulations that reduced spreadability [24].

For LhN and NMP formulations, nearly complete wetting of the glass slide was observed due to their relatively low viscosity. Additionally, the excellent miscibility of aprotic solvents, like NMP, with the aqueous phase of agarose resulted in notably low contact angles on the wet agarose surface. However, formulations such as LhB, LhC, LhA20, LhD15, and LhD20 exhibited higher contact angles on the agarose surface compared to the glass slide. The difference in contact angle values between the two surfaces was statistically significant with a *p*-value < 0.05, as shown in Table 2.

The higher contact angles observed on the agarose surface are attributed to the sufficient concentration of cellulosic esters in these formulations, which induced apparent phase inversion upon solvent exchange. This transition from a solution to a semisolid or solid-like state on the wet agarose surface hindered spreadability [8,16]. Formulations with higher cellulosic ester content were more prone to phase inversion, resulting in greater contact angles on the wet agarose surface. This observed pattern is consistent with earlier findings on in situ matrix systems based on nitrocellulose and borneol [16,17]. Although the developed in situ matrices with a high polymer content exhibited higher contact angles, these values remained below 60°, indicating good wettability on the tested surfaces [8,14].

### 3.3. Mechanical Property

The toughness of the formulations after complete transformation due to solvent removal in agarose wells was assessed using the compression mode of a texture analyzer. Additionally, the adhesion force was determined based on the displacement of the instrument’s probe. The mechanical properties of in situ matrices are crucial following phase inversion, as they must endure jaw movements and maintain their shape within the periodontal pocket [13]. Upon contact with the agarose, the solvent of formulation diffused out, while the aqueous agarose gel induced phase inversion, transforming the water-insoluble cellulosic ester polymers into a gel and subsequently into a solid matrix. The maximum compression and adhesion forces are presented in Table 2. These values were achieved solely for transformed matrices containing 15% and 20% of the cellulosic ester polymers. Conversely, formulations with 5% and 10% cellulosic ester polymers exhibited insufficient firmness after solvent removal, rendering them unresponsive to the test. This highlights the necessity of an adequate amount of intermolecular polymeric chains within the transformed matrices to ensure sufficient structural toughness [25].

Notably, the transformed matrices with 20% polymer content exhibited a significantly higher maximum compression force compared to those with 15% polymer content (*p* < 0.05). A similar dependence of polymer concentration on mechanical properties has been previously reported for transformed nitrocellulose-based in situ matrices [16]. Both the maximum compression force and the adhesion force show an upward trend with increasing polymer concentration, suggesting that the matrix becomes more robust due to enhanced solidification due to solvent removal from the formulation containing sufficient entangled intermolecular interaction between polymeric chains.

Among the tested polymers, matrices formulated with CAB type B displayed greater hardness than those with CAB type A, while those with CAP type C demonstrated higher hardness compared to CAP type D, with a *p*-value < 0.05. The observed differences in hardness also correlated significantly with the molecular weight of the polymers. Regarding adhesion force, matrices containing 20% polymer showed significantly higher values than those with 15% polymer content. Similarly, matrices formulated with CAB type B exhibited higher adhesion forces compared to those with CAB type A, and CAP type C matrices demonstrated superior adhesion forces relative to CAP type D matrices. Typically, longer polymer chains provide greater tensile strength and overall durability, as more force is required to break the chains [25,26]. At lower molecular weights, the polymer chains are loosely connected by weak van der Waals forces, allowing easier chain movement and resulting in reduced strength, despite the presence of crystallinity [26]. Conversely, polymers with higher molecular weights have longer chains that become entangled, thereby increasing the matrix strength.

### 3.4. Matrix Formation

**Figure 2:**
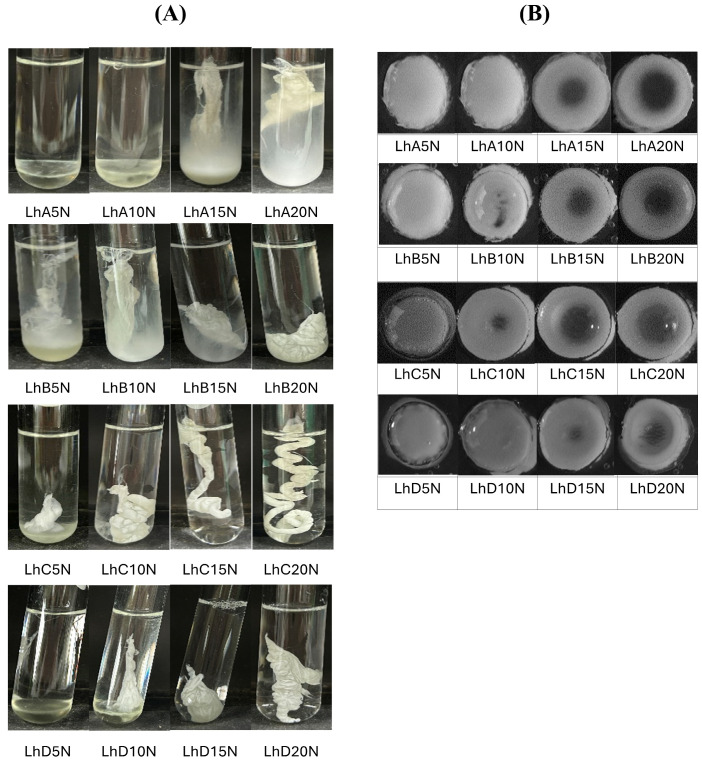
Change in gel formation of Lh-loaded CAB- and CAP-based in situ matrices and control formulations in PBS (pH 6.8) (**A**) and agarose wells at 5 min (**B**).

The phase transformation of the prepared formulations triggered by the aqueous medium was observed after injection into the simulated crevicular fluid in a periodontal pocket (PBS, pH 6.8), as depicted in the photographs in Figure 2A. Formulations without polymer addition, such as LhN and NMP, were compatible with this medium and showed no phase separation. Formulations with low concentrations of CAB and CAP, including LhA5N, LhA10N, and LhD5N, remained in their solution state and settled at the bottom of the test tube.

Some formulations, such as LhA15N, LhA20N, LhB5N, LhB10N, and LhD10N, exhibited partial phase transformation into an opaque, dispersed mass within 5 min of injection into PBS (pH 6.8) (Figure 2A). In contrast, formulations with higher polymer content, including LhB20N, LhC15N, LhC20N, LhD15N, and LhD20N, underwent rapid phase transformation upon exposure to this buffer, resulting in the stabilization of in situ matrices. This transformation triggered gel formation, which gradually developed into a solid matrix, influenced by the retained solvent content over time [8,16].

A cloudy mass resembling a “skin” initially formed around the formulation, which then underwent phase transformation, advancing towards the inner liquid core. Adequate polymer content is critical to controlling drug release; however, excessively viscous formulations may hinder injection through a needle. For CAP type C, a higher polymer content led to a rapid phase inversion, forming a firm, opaque mass followed by a twist-like matrix structure (Figure 2A). The high molecular weight of this polymer significantly accelerated phase inversion and enhanced the toughness of the resulting matrix. In practice, the swift transformation into a gel and matrix polymer can minimize the burst release of the drug. However, excessively rapid phase transformations, such as LhC15N and LhC20N, could lead to the formation of inappropriately shaped masses at the target site. An appropriate rate of phase transformation is essential to ensure the formulation’s ability to compromise its shape and adhere properly to the periodontal pocket. Therefore, achieving an optimal balance between viscosity and polymer concentration is crucial for developing injectable in situ matrix formulations capable of ensuring sustainable drug release.

The cross-sectional view of the matrix formation in the agarose hole at 5 min is displayed in Figure 2B. Formulations containing 5% and 10% polymer exhibited a fully or nearly fully dispersed opaque mass within the agarose cavity. This behavior can be attributed to the rapid removal of the solvent, which facilitated polymer inversion into an insoluble mass through interaction with the aqueous phase. Additionally, formulations with higher polymer concentrations showed an opaque ring surrounding the rim of the agarose. These separated polymeric masses acted as an effective barrier, inhibiting further phase inversion of the formulation’s inner components.

This cross-sectional observation enhances the understanding of the previously described initial skin formation around the formulation. The dense polymeric barrier formed is critical for enabling controlled drug delivery. The initial swift inward diffusion of the aqueous phase from the agarose triggers polymeric phase separation, regulating the outward diffusion of the entrapped drug along with the solvent. The high aqueous insolubility of the cellulosic ester polymers further accelerates this phase inversion. The relatively rapid transformation of the formulation upon injection improves the practicality of periodontitis treatment, enhances patient compliance, and minimizes leakage from the periodontal pocket [27].

### 3.5. Microscopic Interfacial Change

**Figure 3:**
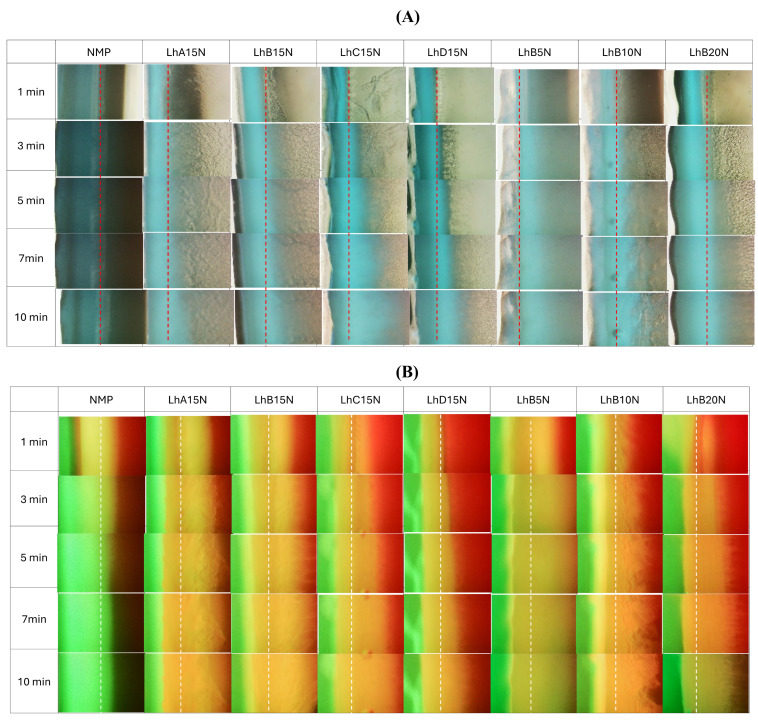
Interface interaction behavior of Lh-loaded CAB- and CAP-based in situ matrices (**right**) with agarose gel (**left**) (**A**); interface interaction between 0.4 µg/mL of sodium fluorescence-loaded agarose gel (**left**) against 2 µg/mL of Nile red-loaded Lh-loaded CAB- and CAP-based in situ matrices (**right**) (**B**) at a magnification of 40× (dash line is the initial interface).

The microscopic images illustrating interfacial changes after contact between the agarose rim (left) and the formulations (right) are depicted in Figure 3A. This experiment aimed to simulate the interaction at the boundary where the formulation meets the biological fluid at the target site [8,28]. Formulations containing 15% CAB and CAP were evaluated and compared. The formulations utilizing CAB type B exhibited relatively easier injectability and a moderate rate of matrix formation. Consequently, these were selected for microscopic interfacial analysis. After the initial phase at 1 min, no notable changes were observed upon the interaction between NMP and agarose. However, all formulations containing 15% polymer exhibited the formation of a gray matrix mass. The matrix characteristics differed between CAP- and CAB-added formulations, potentially due to variations in the substituent groups on their polymeric chains. Specifically, bubble-like droplets were observed in CAB-added formulations, a phenomenon previously reported for lauric acid-based in situ matrices [28], attributed to the rapid solvent movement generating droplets prior to stagnant matrix formation.

The aqueous phase from agarose induced phase transformation in the formulations, resulting in matrix formation driven by polymer phase separation. Theoretically, the energy at the boundary of immiscible substances can drive nucleation and subsequent crystallization of polymers through a thermodynamic mechanism [29]. Thus, crystallization can occur not only at external interfaces, such as those between agarose and the initial crystal, but also at the interfaces of immiscible liquids [28]. Formulations with lower CAB content (e.g., LhB5N) exhibited only a dispersed, thin mass over time, likely due to the insufficient polymer content required to entangle and form a continuous dense matrix, whereas higher CAB content led to thicker matrices. Thus, sufficient polymer content was essential to achieve denser and more pronounced matrix formation for controlled drug release [14]. The matrix-forming materials, including fatty acids, ibuprofen, and borneol-based in situ matrices, similarly experienced interfacial phase separation, resulting in the formation of a thicker crystalline mass that grew progressively over time in a concentration-dependent fashion [8,16,28].

The fluorescent tracking of the solvent and aqueous phase movement was undertaken using a hydrophilic compound, sodium fluorescein, and a hydrophobic compound, Nile red, with the results displayed in Figure 3B. Sodium fluorescein, a dye that emits green fluorescence in aqueous environments, undergoes quenching in organic solvents, leading to a black background. In contrast, Nile red, a lipophilic dye, emits red fluorescence in lipophilic substances and organic solvents, but it undergoes quenching in water, resulting in a black background as well [30,31].

Figure 3B illustrates the microscopic changes at the phase interface between sodium fluorescein-loaded agarose gel and Nile red-incorporated formulations. In the control group, exemplified by NMP, rapid miscibility between the plain solvent and the aqueous phase of the agarose gel resulted in a noticeable loss of red fluorescence. The increased polarity of the mixed solvent likely reduced the solubility of Nile red, causing fluorescence quenching and a subsequent decrease in red fluorescence intensity. At the same time, the bright green fluorescence of sodium fluorescein was observed to enter the NMP, likely attributable to its high affinity with aqueous phase.

LhB15N showed reduced retention of red fluorescence compared to LhC15N and LhD15N, whereas the Nile red fluorescence in LhB5N seemed to quench progressively over time. The expanded yellowish band observed might represent the formation of a cellulosic ester matrix, which likely inhibited the ingress of the aqueous phase from the agarose gel and slowed the removal of NMP from the formulation. The persistence of red fluorescence suggests that the formed matrix effectively hindered drug diffusion outward from the formulation containing NMP.

Formulations containing up to 15% cellulosic ester polymer effectively preserved fluorescence colors (Figure 3B), indicating that an appropriate polymer concentration promoted matrix formation upon contact with agarose. This matrix formation reduced NMP penetration into the agarose and limited the ingress of the aqueous phase into the formulations. Generally, higher viscosity tends to reduce the diffusion rates of solvents and drugs [32], and the diffusion rate can be modulated by altering the polymer concentration in the solution [33].

The increased firmness of the cellulosic ester polymer matrices, as mentioned in mechanical properties, enabled a decelerated solvent exchange. The tracking movement of these dual fluorescent probes was instrumental in observing the dynamic interactions between the solvent and aqueous phases, influenced by the formation of the polymeric in in situ forming systems. The LhB15N and LhB20N formulations exhibited characteristics that highlight their potential as promising candidates for in situ matrix dosage forms designed for localized drug delivery to periodontal pockets. These formulations demonstrated ease of injectability, rapid and stable matrix formation, and effective retardation of solvent exchange. These characteristics make LhB15N and LhB20N especially promising candidates for further drug release studies, particularly when compared to the other formulations.

### 3.6. In Vitro Drug Release and In Vitro Degradation

**Figure 4:**
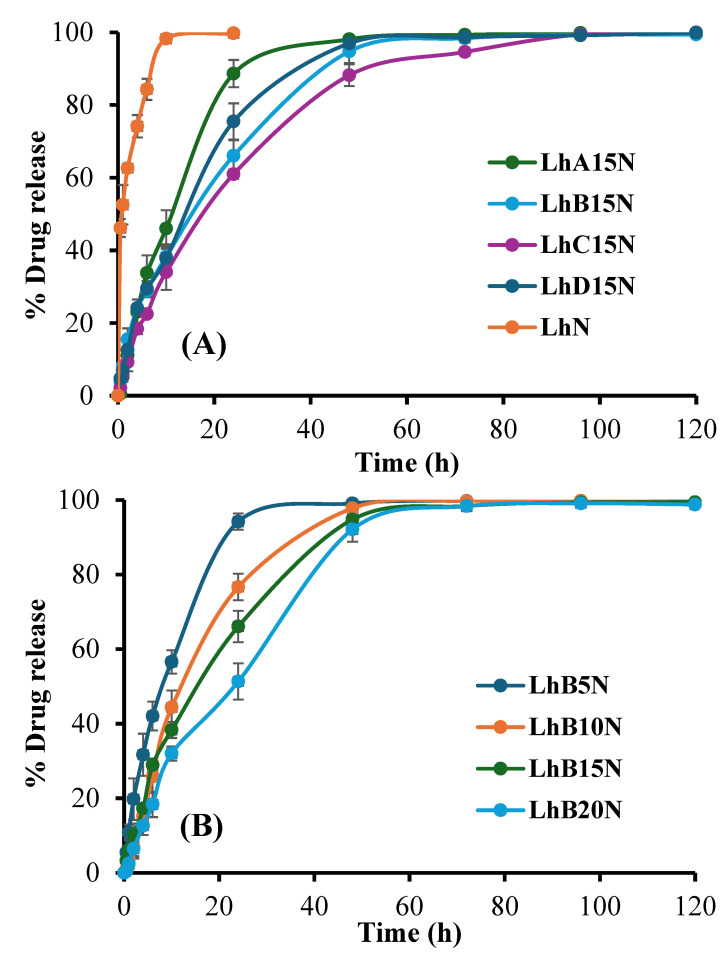
Release profile of Lh from various types of polymers (**A**) and various concentrations of polymer B (**B**) added in situ, forming matrix formulations in PBS (pH 6.8) (*n* = 3).

The drug content for formulations LhN, LhA15N, LhB15N, LhC15N, LhD15N, LhB5N, Lh10BN, and LhB20N was determined to be 98.71 ± 1.27%, 97.44 ± 1.03%, 102.41 ± 0.72%, 99.41 ± 0.83%, 103.61 ± 1.16%, 99.17 ± 0.31%, 101.74 ± 2.61%, and 97.41 ± 1.03%, respectively. The findings suggest that close to 100% drug content was effectively attained through simple continuous mixing until clear solutions formed, without requiring heat or intricate preparation methods. The cup method was utilized to replicate drug release from periodontal pockets in an in vitro setting. Saliva typically maintains a near-neutral pH of approximately 7.06 under normal oral conditions. However, in patients with chronic periodontitis, the average oral pH tends to decline to approximately 6.85 [34,35]. Accordingly, release testing for both the control solution (LhN) and the test formulations was performed in PBS (pH 6.8), aligning with the conditions employed during the gel formation assessment. The cumulative release profiles of Lh over 120 h of selected formulations and LhN are illustrated in Figure 4.

The rapid drug release observed in LhN was attributed to its unrestricted diffusion into the release medium. Among the tested formulations, LhC15N, which incorporated higher molecular weight CAP, demonstrated a more pronounced prolongation of drug release compared to LhB15N, LhD15N, and LhA15N, respectively, as illustrated in Figure 4A, attributed to its elevated viscosity and the toughness of the transformed matrix [36,37,38].

Additionally, increasing the polymer content of CAB type B significantly enhanced drug release retardation, as demonstrated in Figure 4B. Upon contact with the release medium, Lh diffused gradually through the cellulosic ester matrix. Important polymeric attributes, including the transformation rate, matrix toughness, polymeric network density, and porosity, were identified as critical factors influencing the drug release profile [17,24]. Beyond the increased viscosity described earlier, which slowed both drug diffusion and release, the phase inversion into a matrix structure played a central role in sustaining drug release into the surrounding fluid. The prolonged release of doxycycline hyclate over a period of one week has been achieved using solvent removal-based in situ forming systems, in which small molecules, such as ibuprofen and borneol, serve as matrix-forming agents [8,16]. An increased amount of nitrocellulose in in situ forming systems containing salicylic acid and levofloxacin HCl significantly extended the release duration and enhanced permeation through porcine buccal mucosa and skin [17]. Previous studies have also highlighted the role of CAB in delaying the diffusion of moxifloxacin HCl and benzydamine HCl from in situ matrices [39,40]. Therefore, increasing the polymer content led to a rise in formulation viscosity and matrix toughness, which in turn contributed to a more effective extension of drug release duration. The dosage form underwent rapid solidification upon contact with PBS, consistent with earlier findings on gel formation, thereby enabling effective modulation of drug release.

The formation of a cellulosic ester matrix proved its efficacy in minimizing the initial burst release while facilitating sustained drug delivery. Its capability to regulate the release of dual drugs has been previously reported [31]. For comparison, commercially available local drug delivery systems, like Atridox^®^, commonly used by dentists for periodontitis treatment, are formulated to provide sustained antibiotic release over a period of one to two weeks [35]. Moreover, cellulosic ester polymers, such as CAB and CAP, are recognized for their biodegradability, biocompatibility, and non-toxicity [36], highlighting their potential as bioplastic materials for use as matrix-forming agents in in situ drug delivery systems.

Estimating kinetic parameters and evaluating goodness of fit through profile fitting provides meaningful insights into the underlying release mechanisms [13]. These parameters, determined using five mathematical release models, are summarized in Table 3. The goodness of fit was assessed by comparing higher values of the R^2^, lower AIC, and higher MSC values. Most drug release profiles aligned well with a first-order equation, indicating an anomalous drug release pattern. This observation corresponded to the n-value estimated using the Korsmeyer–Peppas equation, which exceeded 0.5, signifying that the release rate depended on the drug concentration remaining in the dosage form [37].

Anomalous transport denotes a mixed mechanism involving both diffusion and erosion, aligning with the observed matrix swelling and erosion behaviors of the formulations [38]. For LhB15N, the release profile followed Higuchi’s model, indicating a diffusion-controlled mechanism attributed to the hydrophobic matrix of cellulose acetate butyrate [29,40]. Fickian diffusion, commonly seen in sustained drug delivery systems, takes place when the matrix encapsulates the drug and controls its release. Initially, a faster release rate is observed, which gradually slows down as the diffusion path lengthens due to saturation of the surrounding fluid [41].

The comparison of k1 and k2 values obtained from fitting the release profile to the Peppas–Sahlin model was used to determine the drug release mechanism [42]. A higher k1 value indicates that diffusion is the dominant process, whereas a higher k2 suggests that polymer relaxation or heterogeneous erosion governs drug release. Occasionally, negative k values may appear, serving as compensatory terms to optimize the data fit. However, these negative values should not be considered when interpreting the release mechanism based on k value comparisons from the Peppas–Sahlin model [42]. From Peppas–Sahlin’s model fitting to the LhB15N release profile, k_1_ was superior to k_2_; thus, the ratio of the Fickian diffusion mechanism was superior to the Case II relaxation mechanism [42]. By comparison, the release rate (k1) from profile fitting to a first-order equation of LhB5N, LhB10N, Lh15BN, and LhB20N was 0.094, 0.055, 0.051, and 0.033 h^−1^, respectively. Notably, the Lh release rate decreased when the content of CAB type B in the formulation increased. The release rate of antibiotic drugs, such as doxycycline hyclate, from the in situ forming system that consisted of a single matrix-forming agent such as Eudragit^®^ L [14], nitrocellulose [17], and saturated fatty acids [28] using the cup method decreased when the concentration of the matrix-forming agent was increased. Higher polymer concentrations likely contribute to a more complex matrix structure with stronger intermolecular bonding, leading to a slower drug release rate.

The in vitro degradation percentages for formulations LhA15N, LhB15N, LhC15N, LhD15N, LhB5N, Lh10BN, and LhB20N were determined to be 92.72 ± 3.69%, 91.13 ± 2.26%, 88.61 ± 1.18%, 90.37 ± 2.41%, 100.00 ± 0.00%, 100.00 ± 0.00%, and 87.79 ± 3.78%, respectively. These results indicate a high degradation capacity under in vitro conditions. Formulations containing higher MW polymers and greater polymer content exhibited lower degradation percentages. Complete mass loss was observed for LhB5N and Lh10BN at 120 h, primarily due to the loss of solvents, while structural components from CAB and CAP disintegrated.

Most of the Lh was liberated from the dosage form during the degradation process, as described earlier. The observed degradation was attributed primarily to solvent and drug loss, along with the partial degradation of the solid matrix components. Additionally, the degradation of the fully formed in situ matrix, combined with drug release kinetics, suggests that an erosion mechanism contributed to drug liberation. This erosion process likely explains the suitability of the Peppas–Sahlin model for describing the drug release profile. Furthermore, the drug release behavior largely following first-order kinetics can be attributed to drug dissolution mechanisms in pharmaceutical dosage forms, particularly those containing water-soluble drugs within porous matrices in which the release rate depended on their drug concentration remaining [43]. To gain deeper insights into the drug release mechanisms from cellulosic ester polymer-based in situ forming matrices, additional investigations of the residual structure were conducted using SEM and SRXTM.

### 3.7. Topography from SEM and SRXTM

**Figure 5:**
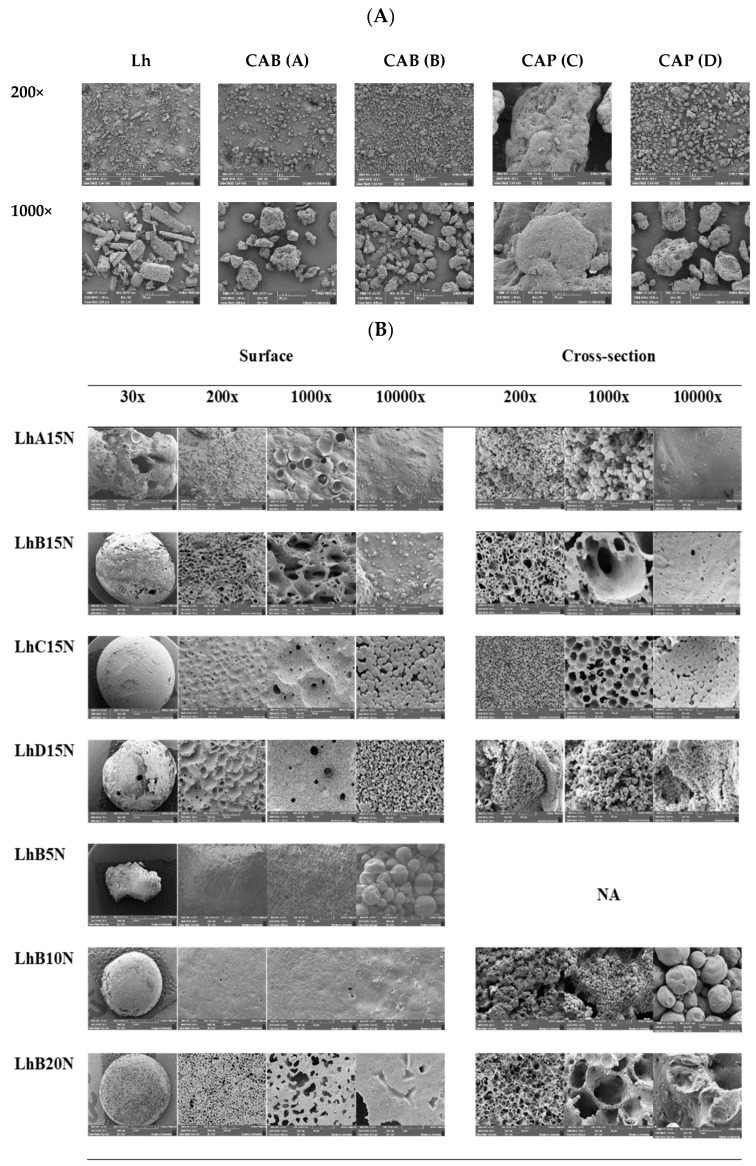
SEM images of the surface view of intact Lh and polymer powder surfaces (**A**) and the surface and cross-section view of different Lh-loaded formulations after release for 120 h (**B**). (NA = not available).

SEM micrographs of the intact raw materials, including Lh, as well as the powders of both CAB and both CAP types, are presented in Figure 5A. The Lh powders displayed a crystalline structure in the form of elongated rectangular bars with varying sizes and dimensions. Among the materials, CAP (C) exhibited a significantly larger particle size compared to CAP (D), CAB (A), and CAB (B), in that order. Notable porosity was observed on the surface topography of CAP (D), CAB (A), and CAB (B) powders, whereas CAP (C) appeared as a large, dense mass with a slightly porous surface.

SEM micrographs of Lh-loaded formulations containing 15% polymer content after a 120 h release study, captured at low magnification (30×), are shown in Figure 5B. For LhA15N, a distorted and irregular shape with large holes was observed, consistent with partial phase transformation during gel formation, as previously noted. This irregularity may indicate structural instability in the transformed matrix following solvent removal.

The remnants of LhB15N and LhD15N displayed porous surfaces with some holes in their structures. In contrast, LhC15N exhibited a relatively dense and smooth surface with only a few pores. Additionally, cracks were visible on the surface of the LhD15N remnant. The film-forming capability of these polymers is largely influenced by the number of hydroxyl groups, while the semi-crystallinity of the films depends on the type and positioning of side groups along the cellulose backbone [3]. Signs of possible cholesteric ordering in the films may be attributed to the increased presence of hydroxyl groups, which disrupt the helix arrangement, while overall structural order is predominantly influenced by substitution patterns and secondarily by molecular weight [44]. As a result, the diverse morphologies observed in the transformed matrices reflect the unique characteristics of each cellulosic ester polymer.

Detailed observations of the surface and cross-sectional views of remnants at higher magnifications are presented in Figure 5B. The type of cellulosic ester appeared to influence the structural characteristics of the remnants. CAB-based remnants exhibited relatively smooth pore walls, while CAP-based remnants exhibited distinctive, rough inner pore walls, particularly noticeable at 10,000× magnification. Although the surface of the LhA15N remnant appeared smooth, its inner structure resembled densely packed droplet-like particles, as evident in the cross-sectional view. This unstable structural shape, combined with the large holes observed at 30× magnification, correlates with its rapid drug release. Similarly, the inner structure of LhD15N showed densely packed solid particles, comparable to LhA15N. Previous studies on Eudragit^®^ RS- and Eudragit^®^ L-based in situ forming matrices demonstrated the formation of aggregated, small-sized spherical nanoparticles, resulting in a compact matrix structure [14,45].

The structural defects, such as holes and cracks, observed in LhD15N remnants likely facilitated a higher release rate when compared to LhC15N. In contrast, cross-sectional views of LhB15N and LhC15N showed scaffold-like structures, indicative of polymer phase separation and solvent removal from the formulations [13,14]. The larger pore size observed in LhB15N facilitated faster Lh release compared to LhC15N. The morphological surface and inner structure of the transformed matrices provide valuable insights into the drug release behavior. LhC15N exhibited a relatively homogeneous and uniform structure with small pore sizes, which effectively controlled and prolonged drug release. On the other hand, the remnants of LhB15N, characterized by larger pore sizes and numerous surface pores, suggested enhanced solvent flow and drug diffusion through the stable matrix structure. The incompletely formed structure was found at 30× at the surface of the LhB5N remnant and its cross-section could not be observed owing to its breakage. The rather smooth surface of LhB5 and LhB10N was observed at 200× and 1000×. The inner structure of LhB10N was not a scaffold, whereas the apparent scaffold-like topography of LhB15N and LhB20N was evident. Therefore, sufficient polymer content was required for scaffold formation after solvent exchange. The incorporation of higher concentrations of bleached shellac, ethyl cellulose, and Eudragit RS into in situ matrices has been shown to further sustain the drug’s release with their denser morphological matrices [45]. The notable larger pore size on the surface of LhB15N relied on its swifter release compared to LhB20N.

**Figure 6:**
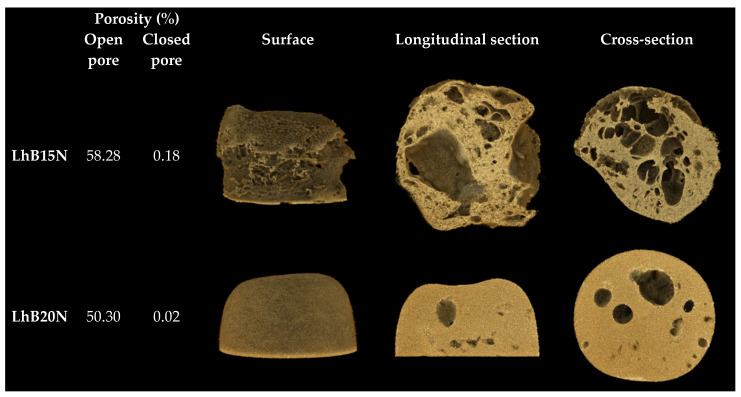
SRXTM and percentage porosity of dried remnants.

SRXTM of LhB15N and LhB20N remnants was displayed with their percentage porosity in Figure 6. The more marked dense structure with a lower density was seen in LhB20N compared to LhB15N. The higher polymer content promoted the smaller pored scaffold of LhB20N and efficient retardation of drug release; nevertheless, its elevated viscosity should be considered for more difficulty of administration via injection. Both % open pore and % closed pore of LhB15N were higher than those of LhB20. An open pore refers to a void that is connected to the external surface of a solid, enabling the diffusion of molecules through the material. In contrast, a closed pore is an isolated cavity within the solid that lacks any connection to the surface and, therefore, does not permit molecular passage [46]. The notable greater open pore than closed pore of these two remnants facilitated the drug diffusion from inner matrices into surrounding release fluid. Therefore, the solvent removal via exchange with the aqueous phase from PBS (pH 6.8) initiated polymer phase separation, which mostly created the open-pored scaffold structure of in situ matrices to modulate drug release.

### 3.8. Antimicrobial Activities

**Figure 7:**
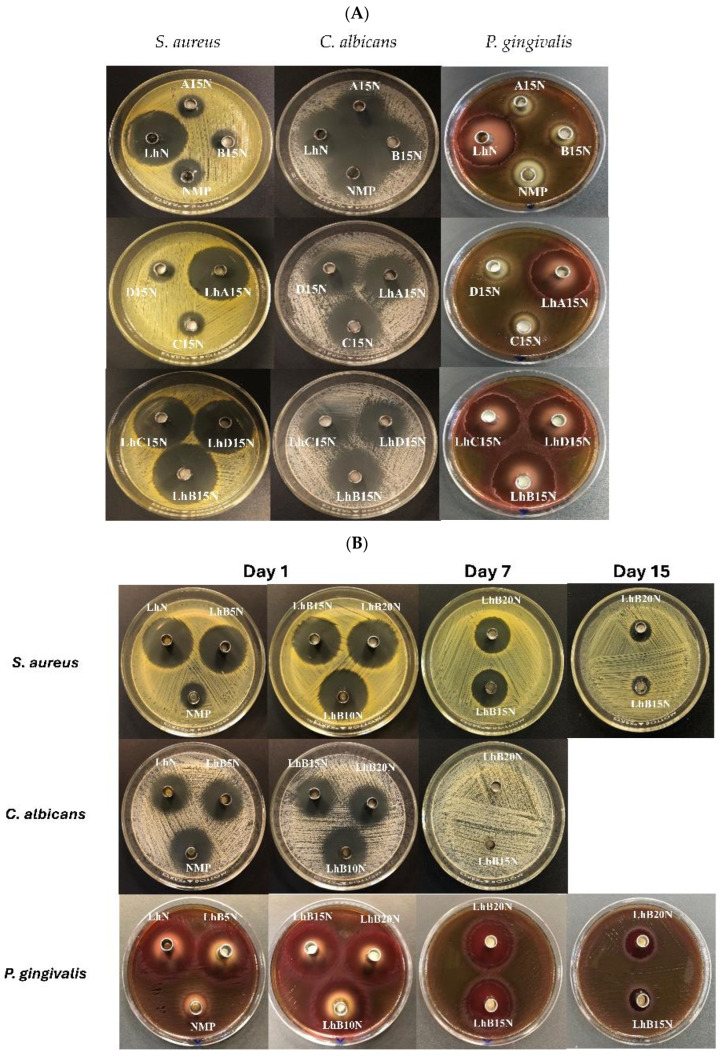
Photographs of the inhibition zone of Lh-loaded CAB- and CAP-based in situ matrices and control formulations (**A**); Lh-loaded CAB type B-based in situ matrix formulations and control formulations on days 1, 7, and 15 (**B**) against three microbes. (NI: no inhibition zone.)

The inhibition zone diameters against three microbial strains for the 15% polymer-loaded formulations and control groups are presented in Table 4A and Figure 7A. Drug-free formulations prepared with 15% of the four polymers (A15N, B15N, C15N, and D15N) served as control groups in this study. Both *S. aureus* and *P. gingivalis* are well-established pathogens linked to dental plaque and periodontal diseases. Among them, *P. gingivalis* is of particular importance in antimicrobial testing due to its role as a key pathogen in anaerobic *Gram*-negative bacterial infections related to periodontitis [9,47]. Similarly, *S. aureus* and *C. albicans* have occasionally been identified in the periodontal pockets of patients with aggressive periodontitis [47].

LhN demonstrated strong antibacterial activity against *S. aureus* 6538 and *P. gingivalis* 33277, surpassing the inhibitory effects of NMP alone. While NMP has been reported to inhibit bacterial growth and effectively suppress *C. albicans* proliferation [48], the degree of its inhibitory action against the three tested microbes is summarized in Table 4. However, the addition of Lh significantly enhanced the inhibition of *S. aureus* 6538 and *P. gingivalis* 33277, as shown in Table 4 and Figure 7. Drug-free formulations with 15% polymer content exhibited slightly lower inhibition zone diameters against the three test microbes compared to NMP alone, likely due to the limited diffusion of NMP from the cellulosic ester matrix into the agar medium. Previous studies have documented the retardation of benzydamine HCl and moxifloxacin HCl diffusion and the resultant reduction in inhibition zone diameters due to obstruction by polymeric matrices [39,40].

Despite this, the inhibition zone diameters of Lh-loaded 15% polymer-based formulations for the two bacterial strains were comparable to those of LhN, indicating that the hydrophilic drug Lh diffused sufficiently through the agar medium to inhibit bacterial growth. However, a noticeable reduction in inhibition zone diameter was observed for the Lh-loaded formulations compared to the drug-free formulations. The inclusion of Lh, which lacks inherent antifungal activity, reduced the NMP content in the formulations, thereby diminishing the antifungal efficacy of NMP against *C. albicans*. Additionally, the polymer matrix further restricted NMP diffusion [8,17]. Nonetheless, this study underscores the utility of NMP as an auxiliary vehicle in the designed dosage forms, enhancing overall antimicrobial activity, particularly antifungal effects.

To assess the sustainability of antimicrobial activity in Lh-loaded CAB type B-based in situ matrix formulations, previously incubated cups from day 1 were transferred to freshly inoculated agar on day 7, and those from day 7 were subsequently transferred to freshly inoculated agar on day 15. The inhibitory zone diameters on day 1 and subsequent changes on days 7 and 15 are reported in Table 4B and Figure 7B. The initial results on day 1 mirrored those previously discussed, with a decreasing trend in the inhibition zone diameter as polymer concentration in the formulation increased. However, the cups containing NMP, LhN, LhB5N, and LhB10N could not be transferred to fresh agar media due to their liquid states (NMP and LhN) or incomplete matrix transformation (LhB5N and LhB10N). In contrast, the solidified matrices of LhB15N and LhB20N, when transferred to fresh agar, exhibited reduced inhibitory diameters against the two bacterial strains, with no inhibition zone observed for *C. albicans*. The absence of antifungal activity after day 1 suggests that the solvent (NMP) was fully removed by day 1, while Lh remained entrapped in the polymeric matrices, gradually diffusing into the inoculated agar over time. This indicates that NMP diffused rapidly and completely from the formulation on the first day, potentially enhancing initial antifungal activity at the target site. NMP, commonly used in pharmaceutical formulations, has low acute toxicity when administered orally, dermally, or via inhalation [49,50]. Atridox^®^, a marketed in situ gel formulation, consists of doxycycline hyclate incorporated into D,L-lactic acid (PLA) dissolved in NMP, which similarly leverages this solvent’s properties [35]. In general, in situ forming matrices intended for drug delivery into periodontal pockets are freshly prepared prior to application, as exemplified by Atridox^®^ [39]. Once administered, the formulation interacts with the moisture present in the periodontal pocket, inducing a phase transition from a liquid to a solid or semisolid state. This transformation enables the controlled release of the drug over an extended period, typically up to seven days [45]. As a result, stability issues in the prepared in situ forming matrix formulations are generally minimal and not considered a significant concern.

Interestingly, LhB20N exhibited a larger inhibition zone on day 7 compared to LhB15N, likely due to its denser matrix, as observed in the SRXTM analysis. This trend became more pronounced by day 15, with a notably greater inhibition zone in LhB20N, reflecting its superior entrapment efficiency and sustained drug release profile. Despite a gradual decline in the inhibition zone over time, the higher polymer content in LhB20N facilitated more prolonged and efficient Lh release. The biocompatibility of CAP and CAB supports their use in biomedical applications. Titanium surfaces coated with CAP have demonstrated non-cytotoxicity and have been found to support effective cell adhesion [51]. Similarly, CAB nanofibers have demonstrated non-toxic properties in cell viability assays for Schwann cells and fibroblasts, highlighting their potential as scaffolds for tissue engineering [52,53]. The LhB15N and LhB20N formulations, particularly, hold promise as injectable in situ matrices for periodontitis treatment due to their controlled drug release profiles and sustained antibacterial activity. The minimum inhibitory concentration (MIC) of levofloxacin against *S. aureus*, *E. coli*, and *P. gingivalis* was reported to be 0.12 µg/mL [54], 0.12 µg/mL [55], and 8 µg/mL [56], respectively. In this study, the total drug release reached a concentration of 43.75 µg/mL (corresponding to 100% release). Therefore, even at an approximately 17.48% release achieved around 2 h, all formulations were able to reach or exceed the MIC required to inhibit *P. gingivalis* as well as the other tested microorganisms. However, further studies, including in vitro cytotoxicity assessments and clinical trials, are essential to validate their therapeutic potential in biomedical applications.

## 4. Conclusions

Lh-loaded in situ matrices designed for the treatment of periodontitis were successfully developed using solvent removal-induced phase inversion, with CAB- and CAP-based polymers as matrix-forming agents. Different concentrations of cellulosic ester polymers, comprising two CAB types and two CAP types, were employed, with NMP serving as the solvent for targeted delivery. Among the formulations, those containing CAB type A exhibited lower viscosity and required less injectability force compared to CAB type B, CAP type D, and CAP type C. The higher molecular weight and concentration of these cellulosic esters significantly increased formulation viscosity but posed challenges for injection. Elevated polymer concentrations increased the contact angle on agarose surfaces due to sufficient cellulosic ester levels inducing noticeable phase inversion. The hardness and adhesive properties of the transformed matrices correlated with the polymers’ molecular weight and concentration. The high aqueous insolubility of these polymers supported phase inversion, forming a semisolid or solid-like matrix in an aqueous environment, although lower molecular weight CAB type A resulted in incomplete matrix firmness. Plain and fluorescent tracking confirmed that matrix mass thickness increased progressively over time in a polymer concentration-dependent manner. The matrix effectively restricted drug diffusion outward along with NMP while also limiting aqueous phase ingress. The LhC15N formulation, containing high molecular weight CAP type A, demonstrated the slowest drug release rate due to its high viscosity and matrix toughness. Additionally, increasing CAB type B content significantly extended drug release duration. High in vitro degradation and release kinetics predominantly following a first-order model suggested drug dissolution and diffusion through porous matrices. The Lh-loaded CAB- and CAP-based formulations showed strong antimicrobial activity, effectively inhibiting periodontitis pathogens and related microbes. Particularly, the Lh-loaded 15% and 20% CAB-based matrices exhibited prolonged bacterial growth inhibition, lasting over 15 days. These two formulations are promising candidates for in situ matrix dosage forms for periodontitis treatment, offering low viscosity suitable for injection, robust gel formation at periodontal pockets, and sustained drug release with potent antimicrobial effects. While cellulosic ester polymers have established safety profiles for various medical applications, further cytotoxicity assessment and clinical studies are essential to confirm the safety and efficacy of these newly developed in situ matrix formulations.

## Figures and Tables

**Table 1:** Compositions of Lh-loaded CAB- and CAP-based in situ matrices and control formulations.

Formulation	% *w*/*w*			Formulation	% *w*/*w*		
	Polymer	Lh	NMP		Polymer	Lh	NMP
**NMP**			100	**LhN**		1	99
**LhA5N**	5	1	94	**LhC5N**	5	1	94
**LhA10N**	10	1	89	**LhC10N**	10	1	89
**LhA15N**	15	1	84	**LhC15N**	15	1	84
**LhA20N**	20	1	79	**LhC20N**	20	1	79
**LhB5N**	5	1	94	**LhD5N**	5	1	94
**LhB10N**	10	1	89	**LhD10N**	10	1	89
**LhB15N**	15	1	84	**LhD15N**	15	1	84
**LhB20N**	20	1	79	**LhD20N**	20	1	79

**Table 2:** Physical properties of Lh-loaded CAB- and CAP-based in situ matrices formulations (*n* = 3).

Formulations	Viscosity (cP)	Injectability (N)	Contact Angle (°)		Compression Force (N)	Adhesion Force (N)
			Glass Slide	Agarose Gel		
**LhA5N**	5.75 ± 0.96	0.086 ± 0.003	9.48 ± 0.17	9.06 ± 0.02	-	-
**LhA10N**	12.91 ± 1.61	0.097 ± 0.07	11.61 ± 0.12	10.22± 0.18	-	-
**LhA15N**	28.76 ± 1.20	0.103 ± 0.013	14.32 ± 0.13	13.48 ± 0.26	5.242 ± 1.15 ^h,l^	0.014 ± 0.008
**LhA20N**	91.84 ± 2.84	0.116 ± 0.011	16.37 ± 0.19	18.27 ± 0.34	9.758 ± 1.12 ^h,m^	0.022 ± 0.005
**LhB5N**	15.02 ± 1.44	0.095 ± 0.004	11.06 ± 0.18	11.17 ± 0.06	-	-
**LhB10N**	53.25 ± 6.95	0.103 ± 0.008	15.51 ± 0.12	17.39± 0.11	-	-
**LhB15N**	230.89 ± 17.12	0.115 ± 0.013	23.22 ± 0.27 ^a^	29.24 ± 0.21 ^a^	17.62 ± 0.78 ^i,l^	0.091 ± 0.064
**LhB20N**	607.15 ± 55.56	0.121 ± 0.014	32.18 ± 0.24 ^b^	41.22 ± 0.27 ^b^	40.64 ± 2.37 ^i,m^	0.143 ± 0.022
**LhC5N**	31.24 ± 0.67	0.107 ± 0.012	17.44 ± 0.26 ^c^	22.36 ± 0.24 ^c^	-	-
**LhC10N**	259.72 ± 4.18	0.116 ± 0.012	23.78 ± 0.28 ^d^	30.31 ± 0.74 ^d^	-	-
**LhC15N**	977.1 ± 95.21	0.124 ± 0.019	32.77± 0.47 ^e^	38.63 ± 0.51 ^e^	28.79 ± 1.72 ^j,l^	0.039 ± 0.03
**LhC20N**	3199.18 ± 64.96	0.132 ± 0.021	39.26 ± 0.67 ^f^	47.61 ± 0.61 ^f^	43.48 ± 1.95 ^j,m^	0.081 ± 0.087
**LhD5N**	18.87 ± 0.09	0.101 ± 0.003	13.44 ± 0.27	13.07 ± 0.04	-	-
**LhD10N**	55.8 ± 1.25	0.112 ± 0.006	20.28 ± 0.36	23.31± 0.10	-	-
**LhD15N**	289.49 ± 15.70	0.120 ± 0.011	27.61 ± 1.32	33.05 ± 0.08	20.97 ± 2.57 ^k^	0.012 ± 0.004
**LhD20N**	673.84 ± 6.09	0.126 ± 0.019	35.67 ± 1.10 ^g^	44.61 ± 0.21 ^g^	42.84 ± 2.07 ^k,m^	0.075± 0.013
**LhN**	4.29 ± 0.27	0.032 ± 0.002	4.06 ± 0.03	3.17 ± 0.02	-	-
**NMP**	3.82 ± 0.04	0.016 ± 0.000	2.11 ± 0.26	2.03 ± 0.18	-	-

The superscripts a–g between columns and h–m in columns represent significant differences (*p* < 0.05); (-) not available.

**Table 3:** Estimate parameters of the release of Lh from various types of polymers and various concentrations of polymer B in situ, forming matrix formulations in PBS (pH 6.8) (*n* = 3).

Formulation	Modeling	Criteria for Model Selection			Kinetic Parameters	
		R^2^	AIC	MSC		
**LhA15N**	Zero order	0.9554	40.6447	2.8234	k0 = 3.970	
	First order	0.9843	33.3498	3.8655	k1 = 0.070	
	Higuchi	0.8963	46.5422	1.9809	kH = 15.372	
	Korsmeyer–Peppas	0.9934	29.2774	4.4473	kKP = 7.637	*n* = 0.775
	Peppas–Sahlin	0.9989	18.5838	5.9749	k1 = −18.864 k2 = 23.781	m = 0.271
**LhC15N**	Zero order	0.9271	38.4334	2.3328	k0 = 2.779	
	First order	0.9924	22.5685	4.5992	k1 = 0.041	
	Higuchi	0.9271	38.4369	2.3323	kH = 17.881	
	Korsmeyer–Peppas	0.9967	18.7378	5.1464	kKP = 6.320	*n* = 0.716
	Peppas–Sahlin	0.9988	13.5157	5.8925	k1 = −8.396 k2 = 13.636	m = 0.281
**LhD15N**	Zero order	0.9157	41.9773	2.1877	k0 = 3.407	
	First order	0.9845	30.1014	3.8843	k1 = 0.056	
	Higuchi	0.9333	40.3368	2.4221	kH = 13.434	
	Korsmeyer–Peppas	0.9949	24.3937	4.6997	kKP = 8.099	*n* = 0.700
	Peppas–Sahlin	0.9949	26.3834	4.4154	k1 = −0.626 k2 = 8.642	m = 0.344
**LhB5N**	Zero order	0.8408	49.6473	1.5517	k0 = 4.443	
	First order	0.9906	29.8253	4.3835	k1 = 0.094	
	Higuchi	0.9659	38.8584	3.0930	kH = 17.881	
	Korsmeyer–Peppas	0.9962	25.4928	5.0024	kKP = 13.079	*n* = 0.625
	Peppas–Sahlin	0.9999	1.5503	8.4229	k1 = −22.709 k2 = 33.825	m = 0.224
**LhB10N**	Zero order	0.9636	37.6915	3.0271	k0 = 3.433	
	First order	0.9868	30.6005	4.0401	k1 = 0.055	
	Higuchi	0.8629	46.9702	1.7015	kH = 13.129	
	Korsmeyer–Peppas	0.9845	33.7021	3.5970	kKP = 5.715	*n* = 0.825
	Peppas–Sahlin	0.9925	30.6083	4.0389	k1 = −16.820 k2 = 19.643	m = 0.284
**LhB15N**	Zero order	0.8189	45.2993	1.4229	k0 = 3.109	
	First order	0.9596	34.7922	2.9240	k1 = 0.051	
	Higuchi	0.9718	32.2757	3.2835	kH = 12.565	
	Korsmeyer–Peppas	0.9954	21.6093	4.8072	kKP = 9.600	*n* = 0.608
	Peppas–Sahlin	0.9961	22.4621	4.6854	k1 = 9.271 k2 = −0174	m = 0.671
**LhB20N**	Zero order	0.9354	36.0975	2.4537	k0 = 2.358	
	First order	0.9858	25.4792	3.9706	k1 = 0.033	
	Higuchi	0.8895	39.8565	1.9168	kH = 9.157	
	Korsmeyer–Peppas	0.9805	29.7217	3.3646	kKP = 4.724	*n* = 0.761
	Peppas–Sahlin	0.9926	24.9658	4.0440	k1 = −17.370 k2 = 19.797	m = 0.236

**Table 4:** Antimicrobial activity of Lh-loaded CAB- and CAP-based in situ matrices and control formulations (A); Lh-loaded CAB type B-based in situ matrix formulations and control formulations on days 1, 7, and 15 (B) against three microbes.

(**A**)			
**Formulations**	**Inhibition Zone Diameter (mm. ± S.D.)**		
	***S. aureus* 6538**	***C. albicans* 10231**	***P. gingivalis* 33277**
NMP	19.0 ± 1.0	34.0 ± 1.7	18.7 ± 0.6
LhN	39.0 ± 1.0	34.0 ± 0.0	34.0 ± 0.0
A15N	17.3 ± 0.6	33.7 ± 0.6	17.3 ± 1.5
B15N	17.7 ± 1.2	33.3 ± 1.5	17.0 ± 1.0
C15N	16.0 ± 1.0	33.7 ± 1.5	17.0 ± 0.0
D15N	17.0 ± 1.0	33.7 ± 1.5	16.7 ± 0.6
LhA15N	41.0 ± 1.0	31.7 ± 0.6	34.3 ± 1.2
LhB15N	40.7 ± 1.2	31.3 ± 1.5	34.3 ± 1.5
LhC15N	40.0 ± 1.0	32.3 ± 1.5	33.0 ± 1.0
LhD15N	39.3 ± 0.6	31.3 ± 1.5	34.7 ± 0.6
(**B**)			
**Formulations**	**Inhibition Zone Diameter (mm. ± S.D.)**		
	***S. aureus*** **6538**	***C. albicans*** **10231**	***P. gingivalis*** **33277**
**Day 1**			
NMP	16.6 ± 0.6	24.7 ± 0.6	13.7 ± 0.6
LhN	34.3 ± 0.6	24.7 ± 0.6	29.3 ± 1.5
LhB 5 N	33.7 ± 0.6	26.3 ± 0.6	28.0 ± 1.7
LhB 10 N	32.7 ± 0.6	26.0 ± 0.0	26.7 ± 0.6
LhB 15 N	33.7 ± 0.6	23.3 ± 0.6	26.3 ± 0.6
LhB 20 N	33.3 ± 1.2	23.7 ± 0.6	26.3 ± 0.6
**Day 7**			
LhB 15 N	26.3 ± 0.6	NI	25.7 ± 0.6
LhB 20 N	27.0 ± 1.0	NI	26.0 ± 0.0
**Day 15**			
LhB 15 N	12.3 ± 0.6	NI	13.7 ± 0.6
LhB 20 N	15.7 ± 1.2	NI	19.0 ± 0.0

NI: no inhibition zone.

## Data Availability

The original contributions presented in this study are included in the article. Further inquiries can be directed to the corresponding author.
